# Moving Stimuli Facilitate Synchronization But Not Temporal Perception

**DOI:** 10.3389/fpsyg.2016.01798

**Published:** 2016-11-17

**Authors:** Susana Silva, São Luís Castro

**Affiliations:** Neurocognition and Language Research Group, Center for Psychology at University of Porto, Faculty of Psychology and Educational Sciences, University of PortoPorto, Portugal

**Keywords:** synchronization, beat, temporal processing, vision, audition, imagery

## Abstract

Recent studies have shown that a moving visual stimulus (e.g., a bouncing ball) facilitates synchronization compared to a static stimulus (e.g., a flashing light), and that it can even be as effective as an auditory beep. We asked a group of participants to perform different tasks with four stimulus types: beeps, siren-like sounds, visual flashes (static) and bouncing balls. First, participants performed synchronization with isochronous sequences (stimulus-guided synchronization), followed by a continuation phase in which the stimulus was internally generated (imagery-guided synchronization). Then they performed a perception task, in which they judged whether the final part of a temporal sequence was compatible with the previous beat structure (stimulus-guided perception). Similar to synchronization, an imagery-guided variant was added, in which sequences contained a gap in between (imagery-guided perception). Balls outperformed flashes and matched beeps (powerful ball effect) in stimulus-guided synchronization but not in perception (stimulus- or imagery-guided). In imagery-guided synchronization, performance accuracy decreased for beeps and balls, but not for flashes and sirens. Our findings suggest that the advantages of moving visual stimuli over static ones are grounded in action rather than perception, and they support the hypothesis that the sensorimotor coupling mechanisms for auditory (beeps) and moving visual stimuli (bouncing balls) overlap.

## Introduction

The advantage of audition over vision in synchronizing with predictable (beat-based) stimuli was held for a long time, based on evidence that synchronization with visual flashes was in many ways poorer than synchronization with beeps (Dunlap, 1910; Repp, 2003, 2005; Patel et al., 2005; Pollok et al., 2009; Birkett and Talcott, 2012), and suggesting that the cognitive system is tuned to guide action with sound rather than sight.

Two recent findings challenged this idea. First, visual stimuli with apparent motion (e.g., moving bars, bouncing balls) seem to outperform static visual stimuli (flashes) in driving synchronization (Hove and Keller, 2010; Hove et al., 2010, 2013a; Gan et al., 2015; Iversen et al., 2015), especially when the direction of stimulus and response movements is compatible (e.g., finger down for bar down, Hove and Keller, 2010; Hove et al., 2010). Second, visual stimuli with motion seem able to match auditory ones in driving synchronization. Earlier research found that synchronization with a moving bar (i.e., a graphical element with a cyclic spatial trajectory) is equivalent to synchronization with a non-optimal auditory stimulus, such as a siren-like sound with waving pitch patterns defining the beat (Hove et al., 2013a). Further research used a bouncing ball with a rectified sinusoidal trajectory and found that moving visual stimuli can even match optimal auditory stimuli: using bimodal videos with congruent (non-distractor) vs. incongruent (distractor) audiovisual stimuli, Hove et al. (2013b) showed that the distractor effects of the bouncing ball on synchronization with beeps are equivalent to the distractor effects of beeps on synchronization with a bouncing ball. Another study found that synchronization with the bouncing ball was as accurate as synchronization with beeps (Iversen et al., 2015), even though it was not the case for all participants. More recently, Gan et al. (2015) increased the realism of the bouncing ball trajectory by simulating gravity and increasing movement smoothness, and they found indistinguishable synchronization performances for balls and beeps. Overall, this research indicated that moving visual stimuli (moving bars, bouncing balls) may drive synchronization more effectively than previously thought. Given that the advantage of moving stimuli was maximal for bouncing balls, we will refer to this finding as the *powerful ball effect*. The powerful ball effect, or the advantage of moving over static visual stimuli, is little more than descriptive, as it merely points out the advantages of a particular type of stimulus in a synchronization task. The general goal of our study was to better understand what this effect says about the human cognitive system. We investigated the extent to which it relates to perception vs. action, and whether it changes when it is based on internal representations rather than on external stimuli.

The reason why visual stimuli require movement to compete with auditory ones is yet to be determined. Is it due to the lower temporal resolution of vision *in perception*, requiring increased reliance on spatial information (Hove et al., 2013a)? Or is it due to constraints pertaining to *sensorimotor coupling* mechanisms (Gan et al., 2015)? Does the cognitive system prefer moving visual stimuli for *processing* or just for *guiding action*? There is currently no answer to these questions. A way to address them is to compare perception with synchronization: if the advantage of moving over visual stimuli stems from resolution constraints in vision, both perception and synchronization should show the powerful ball effect. If it stems from sensorimotor constraints, we should not necessarily expect to see the powerful ball effect in purely perceptual tasks. This second possibility would also support the dissociation between temporal perception and action (synchronization), in line with recent findings (e.g., Fujii and Schlaug, 2013).

The advantage of audition over vision was found earlier in temporal perception than in synchronization (Repp and Penel, 2002). Research on temporal perception faces the paradox that vision has enough resolution to detect fast events but underperforms audition in generating a sense of beat. It seems possible to extract a beat from visually perceived stimuli (Grahn, 2012; Su, 2014), but it is known, for instance, that temporal expectancy is increased in the auditory modality (Pasinski et al., 2016), and that visual beat perception relies on auditory recoding while the reverse is not true (Grahn et al., 2011). Despite the vast literature on modality differences in temporal perception, the advantages of moving over static visual stimuli have been less explored in perception than in synchronization. It is known that infants discriminate moving visual stimuli more accurately than static ones (Brandon and Saffran, 2011), but comparisons between discontinuously moving visual stimuli (rotating bars, changing to non-contiguous spatial positions, as in Hove et al., 2013a) and auditory ones (beeps) showed an advantage of the auditory modality (Grahn, 2012). Bouncing balls (continuously moving stimuli) have not yet been tested as potentially optimal visual stimuli, and the powerful ball effect has not been fully tested in the perceptual domain.

The main goal of the present study was to compare the advantages of moving over static visual stimuli in synchronization vs. temporal perception. Probing the same participants for synchronization and perception allowed us to test possible dissociations between perception and action, to better understand the mechanisms underlying the powerful ball effect, and to advance in the investigation of modality differences in temporal perception. Our challenge was to create perception and synchronization tasks that might capture beat-based timing (in opposition to duration timing, see Teki et al., 2011) and be as similar as possible to one another. In synchronization tasks, the presence of beat-based timing (beat sensitivity) is signaled by anticipation (taps before target beat onsets, see Merchant et al., 2015). Anticipation can be verified in the analysis of tap-beat asynchronies, which should be negative. In perception tasks, evidence of beat-based processing may be captured by participants’ sensitivity to integer multiples or subdivisions of the beat, which imply the beat but do not explicitly focus on it. For instance, Grahn and McAuley (2009); McAuley and Henry (2010), Grahn et al. (2011) developed a paradigm in which participants are probed for the perception of a 600 ms beat unit, after having been exposed to 300 + 300 + 1200 ms intervals. Grube et al. (2010) tested whether participants had a feeling of “wrongness” when exposed to non-integer ratio relationships within a temporal sequence. In our study, participants performed synchronization with isochronous 600 ms intervals, and were later checked for negative tap-beat asynchronies. In the perception task, they were exposed to whole-beats (600 ms) and then probed with 300 ms intervals (half-beats, integer subdivisions) vs. 433 ms or 167 ms (non-integer subdivisions). Participants were asked to detect whether there was something wrong in the sequence.

As a secondary goal, we wanted to know whether and how the powerful ball effect depends on the presence of external stimuli. Do moving visual stimuli maintain the advantage over static ones when participants are forced to rely on internal representations of the beat to synchronize and to perceive the beat? At a general level, answering this question will broaden our knowledge regarding what modulates the powerful ball effect. In the specific context of synchronization, it allows us to test one hypothesis recently raised by Repp and Su (2013): that the mechanisms engaged in the synchronization with beeps and with moving visual stimuli may differ from those used in the synchronization with static visual stimuli. This hypothesis is based on evidence that the transition from synchronization to continuation (continue tapping after stimulus removal) modulates brain activations for beeps and moving visual stimuli, but not for static ones. For instance, the ipsilateral cerebellum, the contralateral dorsal premotor cortex (PMC) and the contralateral ventral PMC are more critical in externally paced than in internally paced synchronization with beeps (Del Olmo et al., 2007; Kornysheva and Schubotz, 2011). Externally paced synchronization with moving visual stimuli relies on the ventral PMC more than internally paced synchronization (Ruspantini et al., 2011). In contrast, there is no difference between external and internal pace in synchronizing with static visual stimuli (Cerasa et al., 2006). This evidence comes from different studies, but, to our knowledge, the effects of pacing modality (external vs. internal) on synchronizing with beeps/moving stimuli vs. static ones have not yet been tested within a single paradigm.

We carried out a behavioral study where a single group of participants performed synchronization and perceptual (forced-choice error detection) tasks, both under stimulus-guided and imagery-guided conditions. In our approach, *stimulus-guided* conditions refer to the presence of external stimuli guiding synchronization and perceptual judgments, and *imagery-guided* conditions refer to stimulus removal after a period of exposure, forcing reliance on an internal beat representation. Imagery-guided synchronization consisted of a continuation phase in a synchronization-continuation task. Imagery-guided perception engaged perceptual judgments of sequences with gaps (no stimuli) in the middle part. In each of the four tasks (stimulus-guided synchronization, imagery-guided synchronization; stimulus-guided perception, imagery-guided perception), participants were tested with optimal vs. non-optimal visual (bouncing ball vs. flash) and auditory (beep vs. siren) stimuli. Thus, we followed the approach of Hove et al. (2013a), in which the powerful ball effect was framed as an interaction between modality and appropriateness, or, in other terms, between modality and continuity (a bouncing ball is appropriate and continuous, a flash is inappropriate and discontinuous; a beep is appropriate and discontinuous, a siren is inappropriate and continuous).

**FIGURE 1:**
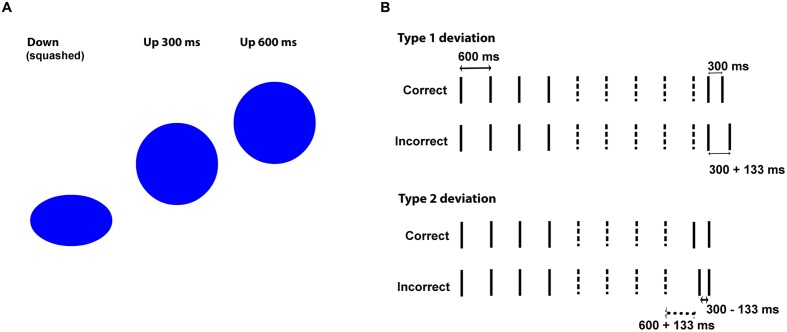
**(A)** Vertical distance moved by the bouncing ball in half-beat (300 ms) and whole-beat (600 ms) intervals. **(B)** Example stimuli in Correct vs. Incorrect versions. Lines represent event onsets. Dashed lines indicate the events deleted to create imagery-based versions. The last two onsets provide the probe interval (the one preserved in imagery-based versions). In incorrect versions with type 1 deviations, only the probe interval is changed, and in those with type 2 deviations both the onset of the first probe-tone and the probe interval are changed.

A necessary step to achieve our goals was to replicate the powerful ball effect. We expected that balls (moving visual) would outperform flashes (static visual) in the stimulus-guided domain; they might even be effective enough to match beeps, but we were not certain of this because we used a linear spatial trajectory with no rectified sinusoidal velocity related to gravity. Previous research suggested that these features might be critical to make balls match beeps (see above), but it is also possible that continuous movement and/or the perception of collision are sufficient to elicit the effect. The studies that showed equivalence between balls and beeps (Hove et al., 2013b; Gan et al., 2015; Iversen et al., 2015) differed from earlier ones in that they used rectified sinusoidal trajectories; however, they were also innovative in the use of continuous movement and a collision point (the imaginary ground, marked with a horizontal line). For instance, the earlier study of Hove et al. (2010) used little or no collision information, and that of Hove et al. (2013a) used only seven steps per beat cycle, which compromises the continuity of the stimulus. In none of these studies did moving stimuli match beeps, and this was attributed to non-rectified trajectories. However, the lack of collision and/or continuity might also have been the cause, and this possibility has not been ruled out by the design of studies showing the powerful ball effect (Hove et al., 2013b; Gan et al., 2015; Iversen et al., 2015). In our study, we use a bouncing ball with a linear trajectory that is totally continuous (30 frames per second, 18 per beat cycle). In order to form the impression of collision, we made the ball squash when hitting the lowest vertical point (**Figure 1**). We were not certain whether this would be enough for balls to match beeps, or if it would just allow balls to match inappropriate auditory stimuli (sirens). Irrespective of how powerful the ball effect would be, our main interest was to find out if the pattern obtained in stimulus-guided synchronization would also be found in the new tasks: imagery-guided synchronization, stimulus-guided perception, and imagery-guided perception.

We reasoned that if the powerful ball effect (interaction between modality and continuity) showed up in synchronization performance but not in perception, this would be evidence that the effect is tied to action in the context of sensorimotor integration, and would support a dissociation between perception and action. If it showed up in both tasks, it would mean that it is tied to perceptual processes. Concerning the effects of stimulus-guided (external pacing) vs. imagery-guided (internal pacing) conditions, we wanted to determine whether they could be observed in synchronization with beeps and balls but not in synchronization with flashes, since this would support the hypothesis of distinct sensorimotor networks for beeps and balls vs. flashes and thus contribute to explain the powerful ball effect. The purpose of exploring the effects of internal vs. external pacing in the perceptual task was exploratory, and its relevance would depend on whether or not a powerful ball effect emerged in stimulus-guided perception.

## Materials and Methods

### Participants

Thirty-four participants (four men) took part in the experiment. They had normal or corrected-to-normal vision, and they were free from hearing, motor, neurological or psychiatric problems. Ages ranged between 19 and 25 years, and schooling between 14 and 18 years. All participants but one were right-handed. Eleven had formal music training beyond elementary school curricula, but only 4 for more than 3 years (1: 4 years; 2: 5 years; 1: 6 years). They all signed informed consent according to the Declaration of Helsinki.

### Stimuli

Visual sequences consisted of videos at 30 frames per second, and auditory sequences of 16 bit, mono audio files at 44.1 kHz sampling frequency. There were short (67 ms) sinusoidal tones (F0 = 450 Hz) in the discrete auditory condition (beeps), waving sinusoidal pitch patterns (F0 between 450 and 337 Hz) in the continuous auditory condition (sirens), short (67 ms) flashes of a blue ball (2.1° diameter) centered over a black background in the discrete visual condition (flashes), and the same ball bouncing on an imaginary ground in the continuous visual condition (a bouncing ball, squashing at the lower point of the trajectory). The spatial trajectory of the bouncing ball was linear.

Sequences for the synchronization task were isochronous and included 50 events (beep onset, siren high pitch, flash onset, squashing ball) with inter-onset-asynchronies (IOIs) of 600 ms (beat length). Sequences for the perception task (**Figure 1B**) tested participants’ abilities in judging whether shorter intervals sounded “right” (correct sequences, 300 ms, integer fraction of the beat) or “wrong” (incorrect sequences, 300 ± 133 ms, non-integer fraction) after being exposed to whole-beat intervals (600 ms). Testing participants’ responses to a beat-compatible interval (300 ms) rather than the beat itself (600 ms) ensured that participants were being tested for beat-based timing rather than duration-based timing (Grahn and McAuley, 2009; Grube et al., 2010; McAuley and Henry, 2010; Grahn et al., 2011). For the perception task, we created eight correct sequences (see Appendix) with lengths ranging from 4200 ms (7 beats) to 6000 ms (10 beats). Stimulus-based correct sequences presented a series of whole beats ending either with two half-beats (stimuli 1–4, see Appendix) or only with one half-beat (stimuli 5–8). Imagery-based sequences were derived from these by keeping the first four beat onsets (three beats) plus the last two onsets (probe interval, last half-beat), and deleting events in-between. Stimulus-based incorrect sequences were derived from stimulus-based correct ones by adding or subtracting 133 ms to either one or two intervals in the terminal part of the sequence. This was accomplished by changing either the last two onsets (one deviant 300 ms interval, stimuli 1–4) or the two onsets preceding the final one (one deviant 600 ms interval and one deviant 300 ms interval, stimuli 5–8). Imagery-based incorrect sequences were derived from stimulus-based incorrect sequences, similarly to what we did to the correct ones.

The 600 ms interval of continuous versions (sirens, balls) was based on time steps of 33.3 ms (one frame in the visual condition), with 18 time steps per beat. The one-beat-length bouncing ball started with a 2-frame (66.6 ms) squashed ball that went up for seven frames while regaining a round shape and returning to the ground in the remaining nine frames. The siren started with a 66.6 ms tone (two time steps), followed by 3.5 descending tones (seven time steps) and 4.5 ascending tones (nine time steps). The 300 ms interval (correct half-beat) was defined by 2 + 3 + 4 time steps (onset + movement + return movement). There were both long (600 ms) and short Inter-Onset-Intervals (300 ms) in the perceptual task, whereas synchronization was implemented with 600 ms IOIs only. Short IOIs are a concern since they seem to degrade visual performance more than auditory performance, at least in synchronization (Repp, 2003). Critically, this is true for bouncing ball conditions, where short IOIs have been associated with unnatural or unrealistic movements (Gan et al., 2015). In order to circumvent these potential deficits in realism, we adjusted the vertical distances moved by the ball as a function of IOIs (shorter IOIs were assigned shorter distances, **Figure 1A**), something that, to our knowledge, has not been done in previous studies. Therefore, the vertical distance moved by the bouncing ball was equivalent to one ball diameter in the 600 ms IOI and to half ball diameter in the 300 ms IOI (**Figure 1A**). The siren oscillated between 450 and 337 Hz in both cases. The vertical distances of the ball and the frequency range of the siren were preserved in the incorrect versions, where four time steps were added or subtracted to the probe interval (distributed as two steps in the ascending direction and two in the descending one). This was done to prevent additional cues in the error detection task.

Half of the incorrect sequences were designed so that the probe interval started on time and had an incorrect length (300 ± 133 ms, *type 1 deviation*, see **Figure 1B** and Appendix), and the other half had a probe interval that started out of time and had also an incorrect length (*type 2 deviation*). In the stimulus-guided domain, type 1 deviations included one incorrect interval while type 2 deviations included two. In the imagery-guided domain, type 1 deviations allowed perceiving an on-time onset followed by an incorrect interval after the empty (imagined) period, while type 2 deviations presented an out-of-time onset followed by an incorrect interval. The four type 1 deviation sequences included two shortened probe intervals (300–133 ms) and two enlarged intervals (300 + 133 ms); the same went for the four type 2-deviation sequences. The reason why we created two deviation types was threefold. First, we needed different sequences for the eight trials and this would be difficult to achieve by varying sequence length only. Second, we wanted to maximize the indices of perceptual performance (discrimination) across conditions, and the response to deviation types could be an additional index. Third, we wanted to explore the mechanisms underlying imagery-guided perception by focusing on the weight of onset-cues (exclusive of type 2) vs. interval-length-cues (increased in type 1). Using onset-cues (detecting off-time onsets, shown in accuracy for type 2 deviations) would mean that participants rely on absolute expectations (when there will be an onset) based on a previously established metrical grid, while ignoring these cues (increased accuracy for type 1 deviations) would mean that participants compare the length of the probe interval with the initial intervals.

All sequences except bouncing balls started with a 200 ms empty interval (silence in auditory sequences, black background in visual flashes). Bouncing ball sequences started with a longer, 600 ms interval featuring the falling ball, which then squashed on the imaginary ground and marked the onset of the first beat. Visual sequences were displayed in a 46 cm-wide monitor, set to a resolution of 1280 pixels × 1024 pixels, with a refresh rate of 60 Hz. Auditory sequences were peak-normalized to 0 dB.

### Procedure

We ran the experiment on E-prime 2^1^. Participants sat 55 cm away from a Samsung Syncmaster 957DF monitor, with a Roland SPD-8 MIDI drum pad sideways (side of the dominant hand). They performed the synchronization task first and then the perception task. In the synchronization task, participants were instructed to use their index finger for tapping along with the stimulus for as long as it lasted (*sensory-guided synchronization*, 30 s or 50 beats), and to keep on tapping after it stopped (*imagery-guided synchronization*) till the experimenter asked them to stop (after 50 taps). They were asked to actively visualize the flash/ball and internalize the sounds when the stimuli were removed. The audio signals generated by hitting the drum pad (audio output) were recorded in an audio file whose onset was locked to the onset of the stimulus. Participants did not hear the audio output from the drum pad. They also wore headphones, which minimized auditory feedback from their own taps. In the perception task, they were first asked to judge whether each of the 16 sequences (8 + 8) was correct or incorrect (*sensory-guided perception*) by pressing key ‘1’ or key ‘2’ on the computer keyboard. We told them that correct versions should sound/look like someone was walking and then started to walk faster, while incorrect versions should sound/look like someone suddenly started to walk with a limp. Afterward, imagery-based versions of the same 16 sequences were presented (*imagery-guided perception*). Sequence presentation was randomized across participants and stimulus types. Participants were given practice trials where they received feedback; they were not informed on the proportion of correct sequences in the experimental trials. They went through all stimulus types in each task before proceeding to the next task.

The four stimulus types (beeps, sirens, flashes, and balls) were ordered in four different ways: beep-siren-ball-flash (auditory-visual, appropriate-inappropriate), siren-beep-flash-ball (auditory-visual, inappropriate-appropriate), ball-flash-beep-siren (visual-auditory, appropriate-inappropriate) and flash-ball-siren-beep (visual-auditory, inappropriate-appropriate). For each of these four orders, we created two conditions in the perception task: one in which the left (‘1’) key meant correct and another where it meant incorrect. Each participant was assigned to one of these eight conditions (four orders × two keys). Half the participants performed auditory first, and the other half visual first.

At the end of the experimental session, participants were given a questionnaire on strategies that they might have used, namely relying on auditory recoding of visual stimuli or vice-versa.

### Data Preprocessing and Statistical Analysis

**FIGURE 2:**
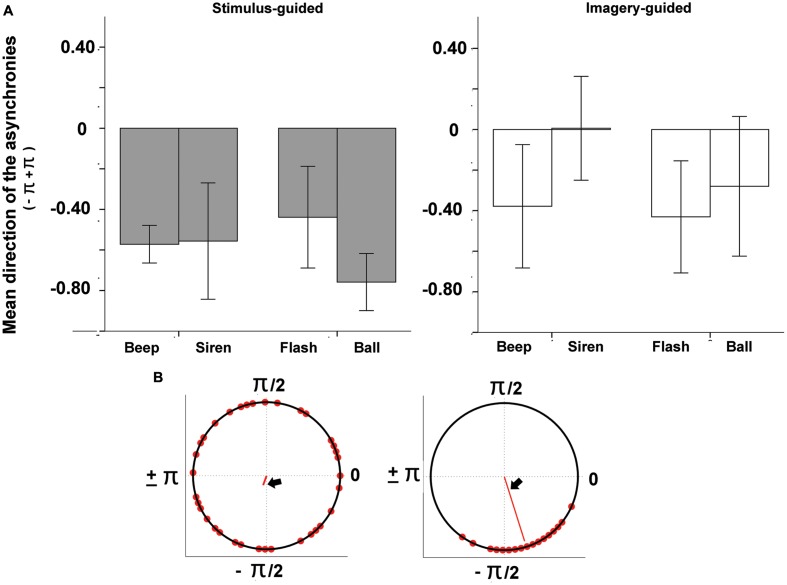
**(A)** Mean direction of tap-beat asynchronies (represented as relative phases) as a function of modality (auditory – beep and siren; visual – flash and ball) and continuity (discontinuous = beep and flash; continuous = siren and ball). **(B)** Example circular plots, where the two different angles indicate different mean asynchronies (both negative; left: -1.92, right, -1.27).

**FIGURE 3:**
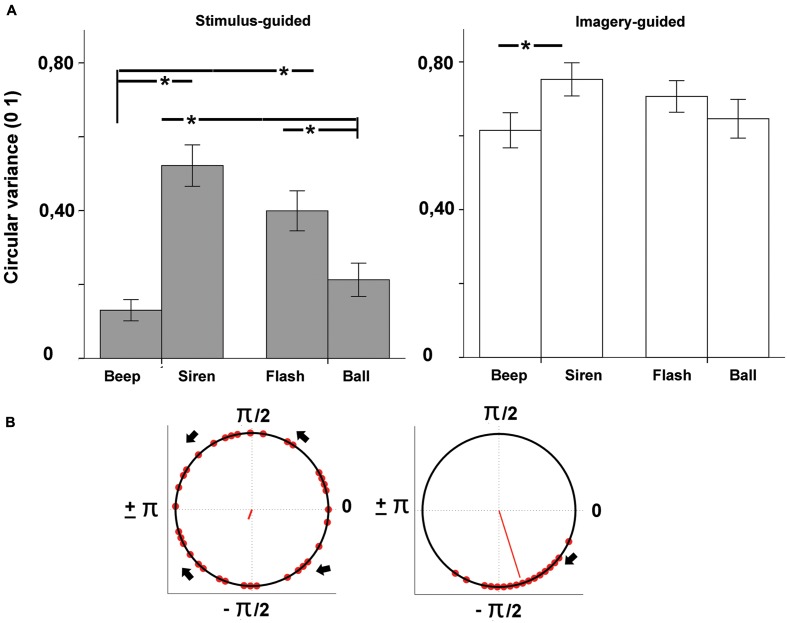
**(A)** Variability of tap-beat asynchronies (circular variance) as a function of modality (auditory – beep and siren; visual – flash and ball) and continuity (discontinuous = beep and flash; continuous = siren and ball). Asterisks indicate significant differences. **(B)** Example circular plots showing high (left, 0.86) vs. low (right, 0.08) circular variances (lower variance indexed by longer red line). High variances tend to be distributed uniformly across the circle as illustrated in the leftmost plot.

The audio files generated by participants’ synchronization were analyzed in Praat^2^. Tap onsets were detected with the function “annotate –to text grid (silences),” which determines the onset and offset of silent vs. sounding periods in the audio files. The first 2 s (four events) of both the sensory-guided and the imagery-guided synchronization tasks were discarded from analysis. Deviant intervals (longer than 1000 ms and shorter than 200 ms) were also discarded. In line with relevant studies in this field (Hove et al., 2013b; Gan et al., 2015; Iversen et al., 2015), synchronization performance was tested for phase-matching (tap-beat asynchronies) with circular statistical methods (Fisher, 1993) as implemented in the Circstats toolbox (Berens, 2009) for Matlab^3^. For each tap, we computed the asynchronies to both the previous and the following beat, and we chose the shorter asynchrony. Asynchronies were then represented in terms of relative phase: each tap was mapped onto a unit circle where a phase of 0 means perfect alignment with the beat, negative values (0 to -pi, or -300 ms, half cycle) indicate anticipation, and positive values (0 to +pi or +300 ms) indicate delay (see **Figures 2B** and **3B**). We computed the mean direction of the asynchronies (function circ_mean, **Figure 2B**) as well as circular variance (function circ_var, **Figure 3B**). Circular variance indexes the variability in tap-beat asynchronies and it may range from 0 (lowest variability in asynchronies) to 1 (highest variability, with uniform distribution around the circle). Measures of asynchrony are not typically used when there is not an external pacing-signal. However, they were necessary in our study, since we needed a common measure to compare stimulus-guided with imagery-guided synchronization. In imagery-guided synchronization, tap asynchronies were computed in relation to virtual beats, which continued the real ones without interruption. The internal generation of virtual beats was explicitly requested to participants (see Procedure). In order to grant a control analysis with a more typical measure of internally paced synchronization, we also computed the mean and variability of Inter-Response-Intervals (IRI, the mean value was the mean deviation from the target interval of 600 ms) for the two domains (stimulus- and imagery-guided). These measures focus on period matching rather than phase matching. We also measured the error correction for period, as indexed by the negative direction of lag 1 autocorrelation for IRIs. The negative value of the lag-1 autocorrelation means that a longer interval tends to be followed by a shorter one, which is often taken as a sign of online error correction (Iversen et al., 2015). Given that the onset of the output audio files was locked to the onset of stimuli, asynchrony (phase) measures could be computed by comparing the timing of taps with the beat structure of stimuli.

Performance on the perception task (discrimination between correct and incorrect) was approached with d-prime measures (Stanislaw and Todorov, 1999). Additionally, we analyzed the effects of deviation type (type 1 vs. type 2) on accuracy for incorrect targets (correct rejections). In all analyses, we used repeated-measures ANOVAs with domain (stimulus-guided vs. imagery-guided), modality (auditory vs. visual) and continuity (discontinuous, continuous) as factors. Deviation type was added when analyzing effects on correct rejections. In case of interactions involving domain, we analyzed stimulus-guided and imagery-guided separately. Significant modality × continuity interactions were further tested with paired-samples *t*-tests, using Bonferroni-corrected significance levels. In order to test the hypothesis that the transition from synchronization (stimulus-guided) to continuation (imagery-guided) has different effects on balls and beeps vs. flashes, we also analyzed the effects of domain on each of the four stimulus types, in case of interactions involving domain. When analyzing the effects of deviation type on correct rejections, we compared type 1 with type 2 deviations in each of the eight conditions. Because some distributions presented deviations from normality, we cross-checked the paired comparisons with non-parametric tests. In the perceptual error detection task, *d*-prime values for each condition were first tested against zero with one-sample *t*-tests.

Even though we had few participants with musical training and training was relatively modest, we wanted to rule out any effects of musical experience. Musical experience is known to influence rhythmic performance (Chen et al., 2007), although it depends on the instrument played by the musician (Krause et al., 2010). To that purpose, we tested if the years of musical training predicted performance in each dependent measure, using linear regression analyses (years of musical training as predictor). Since we had eight conditions per measure (4 stimuli × 2 domains), we used a threshold for significance of 0.0062 (0.05/8). Finally, we tested whether variability in tap-beat asynchronies and IRI variability (synchronization performance) correlated with *d*-prime (perceptual performance).

## Results

### Synchronization

#### Mean Direction of Asynchronies

The mean direction of asynchronies (**Figure 2**) was negative for all conditions except sirens in the imagery-guided domain. Negative values indicated that taps occurred before the beat, and that there was anticipating behavior consistent with beat-based timing. There were no significant interactions involving domain (*p* > 0.059). The interaction between modality and continuity was non-significant (*p* > 0.45), and so were the main effects of domain (*p* > 0.62), modality (*p* > 0.57) and continuity (*p* > 0.74).

#### Circular Variance

The omnibus ANOVA for circular variance (**Figure 3**) showed a significant effect of domain [stimulus-guided < imagery-guided: *F*(1,33) = 102.54, *p* < 0.001, $\eta_{p}^{2}$ = 0.757] and a significant domain × modality × continuity interaction [*F*(1,33) = 12.30, *p* = 0.001, $\eta_{p}^{2}$ = 0.272]. In the stimulus-guided domain, the modality × continuity interaction was significant [*F*(1,33) = 62.23, *p* < 0.001, $\eta_{p}^{2}$ = 0.653]. Comparisons between the four conditions (beep, siren, flash, ball, **Table 1**) showed less variability for beeps compared to sirens and flashes, and for balls compared to sirens and flashes. Beeps did not differ from balls, and sirens did not differ from flashes. In the imagery-guided domain, the modality × continuity interaction was also significant [*F*(1,33) = 9.46, *p* = 0.004, $\eta_{p}^{2}$ = 0.223], but comparisons between the four conditions showed differences between beeps and sirens only. Cross-domain comparisons for each stimulus (beep, siren, flash, ball in stimulus-guided vs. imagery-guided) using an alpha-level of *p* = 0.012 (0.05/4) showed decreased performance for all stimulus types in the imagery-guided domain compared to the stimulus-guided domain [beeps: *t*(33) = -8.81, *p* < 0.001, *d* = 1.45; sirens: *t*(33) = -4.72, *p* < 0.001, *d* = 0.74; flashes: *t*(33) = -5.71, *p* < 0.001, *d* = 0.96; balls: *t*(33) = -7.26, *p* < 0.001, *d* = 1.25]. Cross-stimulus comparisons of the amount of performance decrease (alpha-level of *p* = 0.008) showed that beeps were significantly more prone to performance decline than sirens [*t*(33) = 3.62, *p* = 0.001, *d* = 0.78]; the remaining comparisons yielded non-significant results [beeps vs. flashes: *t*(33) = 2.70, *p* = 0.011, *d* = 0.53; beeps vs. balls: *t*(33) = 0.93, *p* > 0.36, *d* = 0.39; balls vs. sirens: *t*(33) = 2.70, *p* = 0.011, *d* = 0.36; balls vs. flashes: *t*(33) = 1.86, *p* = 0.072, *d* = 0.12; sirens vs. flashes: *t*(33) = 1.14, *p* > 0.026, *d* = 0.26]. The number of years of musical training did not significantly predict circular variance (*p*s > 0.13) for any stimulus type × domain condition.

**Table 1:** Synchronization performance across stimulus types.

	Circular variance						IRI variability (domain collapsed)			Lag-1 autocorrelation (domain collapsed)		
							
	Stimulus-guided			Imagery-guided				
				
	t/Z^a^	d	*p*(t/Z)	t/Z	*d*	*p*(t/Z)	t/Z	*d*	*p*(t/Z)	t/Z	*d*	*p*(t/Z)
Beep-Siren	**-7.17**	**-1.22**	**<0.001**	**-3.24**	**-0.5**	**0.003**	**-**2.18	**-**0.48	0.036	**-**2.57	**-**0.58	0.015
	**-4.85**		**<0.001**	**-3.00**			**-**2.29		0.021	**-**2.35		0.019
Beep-lash	**-5.73**	**-0.93**	**<0.001**	**-**1.81	**-**0.37	0.059	**-3.33**	**-0.71**	**0.002**	**-**2.08	**-**0.41	0.045
	**-4.73**		**<0.001**	**-**1.79			**-4.06**		**0.001**	**-**1.87		0.061
Beep-Ball	**-**0.2.24	**-**0.36	0.032	**-**0.61	**-**0.14	0.543	**-**2.27	**-**0.41	0.030	1.58	0.31	0.122
	**-**1.77		0.077	**-**0.76			**-**2.26		0.023	1.37		0.169
Ball-Siren	**-6.17**	**-0.93**	**<0.001**	**-**2.29	**-**0.34	0.029	**-**1.60	**-**0.35	0.119	**-3.40**	**-0.78**	**0.002**
	**-4.75**		**<0.001**	**-**2.27			**-**0.12		0.898	**-3.00**		**0.003**
Ball-Flash	**-4.45**	**-0.63**	**<0.001**	**-**1.23	**-**0.21	0.227	**-3.08**	**-0.59**	**0.004**	**-3.17**	**-0.60**	**0.003**
	**-4.14**		**<0.001**	**-**0.53			**-3.07**		**0.002**	**-3.02**		**0.003**
Siren-Flash	2.34	0.37	0.026	**-**0.98	0.15	0.331	0.77	**-**0.16	0.444	0.24	0.05	0.806
	2.13		0.033	**-**01.03			1.66		0.096	0.26		0.791


*^a^Wilcoxon sign-rank test. Bolded values indicate significant differences.*

#### Mean Deviation from Target Interval

The *mean IRI deviation from the target* interval (600 ms) did not change significantly as a function of either modality or continuity (*p*s > 0.20), but there was an effect of domain [*F*(1,33) = 13.39, *p* = 0.001, $\eta_{p}^{2}$ = 0.289], indicating increased deviations in the imagery-guided condition than in the stimulus-guided one.

#### Variability in Inter-Response-Intervals (IRIs)

**FIGURE 4:**
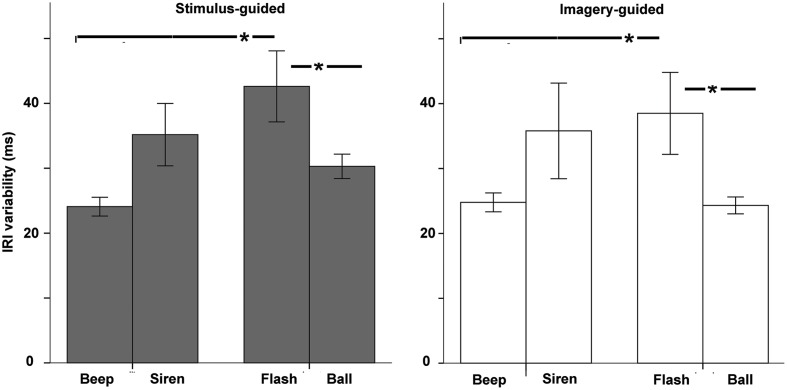
**Variability of the Inter-Response-Interval (standard deviation of tapped intervals) as a function of modality (auditory – beep and siren; visual – flash and ball) and continuity (discontinuous = beep and flash; continuous = siren and ball).** Asterisks indicate significant differences.

The omnibus ANOVA for IRI variability (standard deviation of the produced intervals, **Figure 4**) showed a significant modality × continuity interaction [*F*(1,33) = 12.08, *p* = 0.001, $\eta_{p}^{2}$ = 0.268] and no interaction involving domain (stimulus-guided vs. imagery-guided). Comparisons between the four conditions (beep, siren, flash, and ball) collapsed across domain (**Table 1**) showed significantly improved performance in beeps compared to flashes and in balls compared to flashes. The remaining comparisons were non-significant. Non-parametric (Wilcoxon signed rank) tests confirmed these results. The number of years of musical training did not significantly predict IRI variability (*p*s > 0.19) for any stimulus type × domain condition.

#### Lag 1 Autocorrelation

**FIGURE 5:**
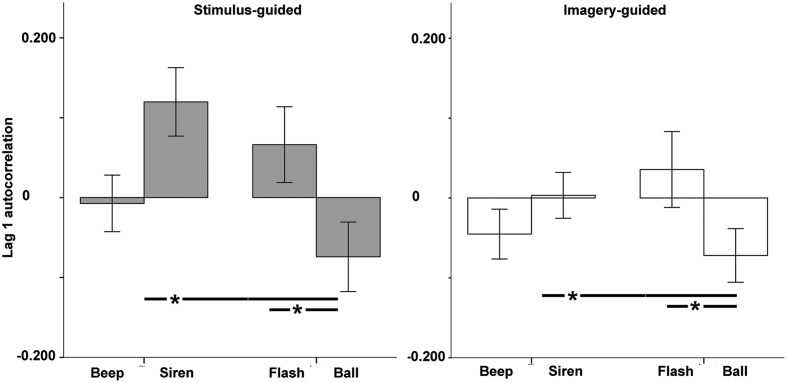
**Lag 1 autocorrelation as a function of modality (auditory – beep and siren; visual – flash and ball) and continuity (discontinuous = beep and flash; continuous = siren and ball).** Asterisks indicate significant differences.

For lag 1 autocorrelation (**Figure 5**), there was a significant modality × continuity interaction [*F*(1,33) = 15.34, *p* < 0.001, $\eta_{p}^{2}$ = 0.317], with no simple effects or interactions involving domain (*p*s > 0.17). Beeps and balls showed negative values (larger for balls), indicating the presence of error correction, while sirens and flashes showed positive ones, indicating persistence. Comparisons across the four conditions (**Table 1**) indicated more negative values for balls vs. sirens and for balls vs. flashes. There were no significant differences between beeps and sirens, beeps and flashes, beeps and balls, or sirens and flashes. Non-parametric tests confirmed these results.

The number of years of musical training did not predict lag 1 autocorrelation any condition (*p* > 0.018), given the Bonferroni-corrected threshold for significance (*p* = 0.006).

### Perception

#### Discrimination

**FIGURE 6:**
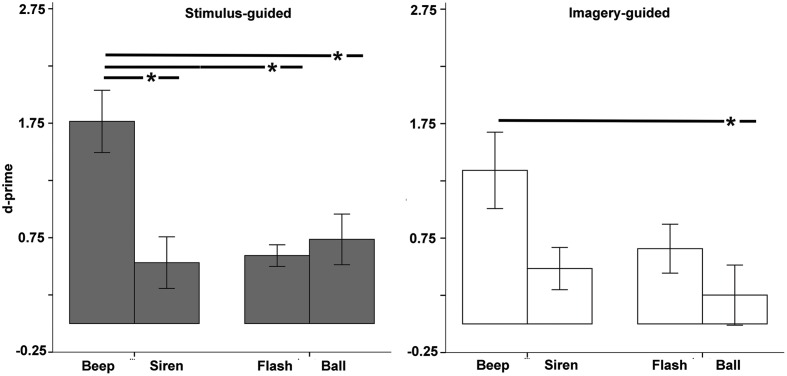
**Discrimination between correct and incorrect sequences (*d*-prime) as a function of modality (auditory – beep and siren; visual – flash and ball) and continuity (discontinuous = beep and flash; continuous = siren and ball).** Asterisks indicate significant differences.

*D*-prime values differed significantly from zero in all conditions (all *p*s < 0.024), except the imagery-guided ball (*p* = 0.344, **Figure 6**). The omnibus ANOVA yielded a significant domain × modality × continuity interaction [*F*(1,33) = 9.22, *p* = 0.005, $\eta_{p}^{2}$ = 0.218, **Figure 6**], and thus we examined stimulus-guided and imagery-guided domains separately.

The stimulus-guided domain showed a modality × continuity interaction [*F*(1,33) = 6.71, *p* = 0.014, $\eta_{p}^{2}$ = 0.169]. Comparisons across the four conditions (**Table 2**) found increased discrimination for beeps compared to the other three conditions. Balls did not differ from flashes or sirens, and flashes did not differ from sirens. Non-parametric tests confirmed this. The imagery-guided domain showed significant modality and continuity main effects [modality: *F*(1,33) = 7.15, *p* = 0.012, $\eta_{p}^{2}$ = 0.178; continuity: *F*(1,33) = 7.04, *p* = 0.012, $\eta_{p}^{2}$ = 0.176]. Beeps elicited increased discrimination compared to balls; no other differences were observed. The number of years of musical training did not significantly predict *d*-prime (*p*s > 0.25) for any stimulus type × domain condition.

**Table 2:** Perceptual performance across stimulus types.

	Discrimination (*d*-prime)					
	
	Stimulus-guided			Imagery-guided		
		
	t/Z	*d*	*p* (t/Z)	t/Z	*d*	*p* (t/Z)
Beep-Siren	**4.19**	**0.78**	**0.001**	2.41	0.53	0.021
	**3.51**		**0.001**	1.78		0.074
Beep-Flash	**3.88**	**0.89**	**0.001**	2.68	0.41	0.011
	**2.98**		**0.003**			0.290
Beep-Ball	**3.52**	**0.68**	**0.001**	**3.55**	**0.60**	**0.001**
	**2.98**		**0.003**	**3.22**		**0.001**
Ball-Siren	0.69	0.15	0.493	-0.79	-0.17	0.433
	1.09		0.274	-1.05		0.574
Ball-Flash	0.56	0.14	0.575	-1.44	-0.28	0.157
	0.87		0.383	-1.74		0.080
Siren-Flash	-0.41	-0.06	0.687	-0.62	-0.14	0.537
	-0.57		0.564	-0.64		0.516


*Bolded values indicate significant differences.*

The questionnaires showed only two reports of modality recoding strategies, where participants mentioned auditory recoding of bouncing balls as auditory stimuli. However, analyses of order effects (auditory first vs. visual first) on discrimination performance in the whole group showed no evidence of recoding (i.e., that visual performance improved when visual stimuli were preceded by auditory ones, see Grahn et al., 2011).

#### Effects of Deviation Type on Correct Rejections

**FIGURE 7:**
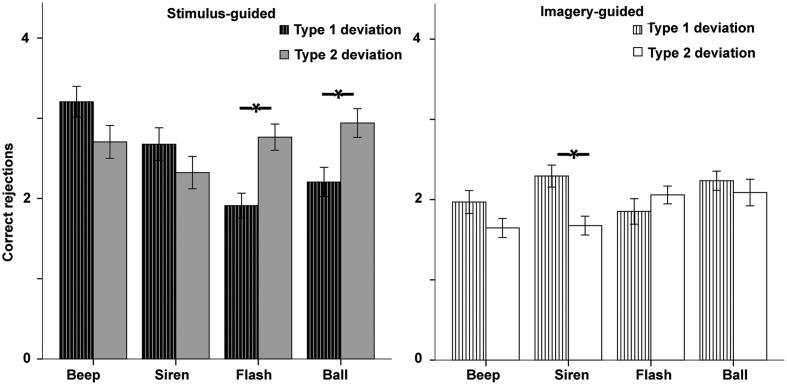
**Accuracy for the two deviation types in incorrect sequences (correct rejections) in the error detection task as a function of modality (auditory – beep and siren; visual – flash and ball) and continuity (discontinuous = beep and flash; continuous = siren and ball).** Asterisks indicate significant differences.

The ANOVA (**Figure 7**) showed a deviation type × domain × modality interaction [*F*(1,33) = 5.79, *p* = 0.022, $\eta_{p}^{2}$ = 0.149]. Deviation type interacted with modality in the stimulus-guided domain [*F*(1,33) = 21.13, *p* < 0.001, $\eta_{p}^{2}$ = 0.390] as well as in the imagery-guided domain [*F*(1,33) = 10.02, *p* = 0.003, $\eta_{p}^{2}$ = 0.233], but there was also a marginal deviation type × continuity effect in the latter [*F*(1,33) = 2.87, *p* = 0.10, $\eta_{p}^{2}$ = 0.080]. Analyses of deviation type effects in stimulus-guided conditions showed that type 2 deviations increased correct rejections in visual stimuli [flashes: *t*(33) = -3.36, *p* = 0.002, *d* = -0.84; balls: *t*(33) = -2.68, *p* = 0.011, *d* = -0.66] but not in auditory ones [beeps: *t*(33) = 1.84, *p* = 0.074, *d* = 0.41; sirens: *t*(33) = 1.32, *p* = 0.195, *d* = 0.30]. In imagery-guided conditions, type 1 deviations increased correct rejections in auditory continuous stimuli [sirens: *t*(33) = 3.45, *p* = 0.002, *d* = -0.77] but not in the other three [balls: *t*(33) = 0.81, *p* = 0.419, *d* = 0.16; beeps: *t*(33) = 1.77, *p* = 0.086, *d* = 0.42; flashes: *t*(33) = -1.26, *p* = 0.214, *d* = -0.25].

### Correlations between Synchronization and Error Detection

Participants’ sensitivity to error in the perceptual task (*d*-prime) did not correlate either with their circular variance (*p*s > 0.036) nor with IRI variability (*p*s > 0.14) for any of the eight domain (2) × stimulus (4) conditions.

## Discussion

Our main goal was to find out whether the recently discovered advantage of moving over static visual stimuli in sensorimotor synchronization (the powerful ball effect) extends to purely perceptual tasks. We wanted to better understand the cognitive underpinnings of the powerful ball effect, test for dissociations between perception and synchronization, and advance our knowledge on modality effects on perception. In order to accomplish the main goal, we compared synchronization performance with performance in a perceptual task, both involving moving (bouncing balls) and static visual stimuli (flashes). Optimal (beeps) and non-optimal (sirens) auditory stimuli were also involved in the comparison, so as to determine if moving visual stimuli were effective enough to match beeps — the most effective stimulus known till now.

The results pointed to a dissociation between synchronization and perception. In synchronization, balls not only outperformed flashes, but they also matched beeps in all measures of variability and error correction. In the purely perceptual task, there was no powerful ball effect: moving visual stimuli (balls) were as insufficient as static ones (flashes) to facilitate discrimination between correct and incorrect versions, and both were less efficient than beeps. In addition, the effects of deviation type (type 1 vs. type 2) on accurate rejections did not change according to what could be expected in a powerful ball scenario, which should present a modality × continuity interaction (balls dissociate from flashes and beeps from sirens). Instead, there was a modality effect: both flashes and balls showed more correct rejections for type 2 deviations than for type 1, while auditory stimuli did not.

The dissociation between synchronization and perception rules out the possibility that movement serves to compensate for temporal-resolution problems in vision. If this were the case, we should have seen the powerful ball effect in the perceptual task too. Rather, the dissociation seems to suggest that the powerful ball effect is linked to motor action in the context of sensorimotor synchronization. The possibility that the powerful ball effect is specifically related to action is in line with evidence that synchronization quality depends on the compatibility between the spatial trajectories of response taps and moving visual stimuli, such that synchronization improves when the two share the same direction (Hove and Keller, 2010; Hove et al., 2010).

Concerning temporal perception, our findings expand those of Grahn (2012) who found superior perceptual performance for beeps compared to a discontinuously moving bar. In our study, beeps outperformed a continuously moving ball. Therefore, continuous movement does not seem to be sufficient to make visual stimuli as efficient as auditory ones in perception, at least in the absence of naturalistic kinematics.

Our secondary goal was to examine how the powerful ball effect is modulated by external vs. internal pacing. To that end, we compared stimulus-guided synchronization and perception with imagery-guided synchronization and perception. In synchronization, we had mixed results: asynchrony (phase-matching) measures showed modulations (interactions with domain), while period-matching measures (IRI variability, lag-1 correlation) did not. Given that asynchrony measures are dominant in sensorimotor synchronization studies (Repp and Su, 2013), we will favor these to interpret our findings. We found that the variability of tap-beat asynchronies (circular variance) was lower for beeps and balls than for flashes and sirens in the stimulus-guided domain. In imagery, there was an advantage of beeps over sirens. We focused on the effects of the transition from synchronization (stimulus-guided) to continuation (imagery-guided) on each stimulus, since these were critical for testing the hypothesis that the same sensorimotor coupling mechanisms are recruited by beeps and balls, and these differ from the mechanisms recruited by flashes. We followed the reasoning of Repp and Su (2013), suggesting that similar responses to the transition from stimulus-guided synchronization to imagery-guided synchronization would indicate shared sensorimotor coupling mechanisms (see introduction). Support to this hypothesis would be provided if the performance decline for beeps when entering the continuation phase was equivalent to that of balls, and if both balls and beeps differed from flashes in this regard. While the first condition was observed – beeps and balls were equally affected by stimulus removal, the second was not: even though both balls and beeps were more affected by stimulus removal than flashes, the difference between beeps and flashes was marginal, and that between balls and flashes did not reach significance. Therefore, we found partial support to the hypothesis that beeps and balls recruit the same sensorimotor coupling mechanisms, and that both differ from flashes in this respect. The fact that we used measures of tap asynchrony relative to virtual (imagined) beats, and that we could not control for the timing of the imagined beats was a limitation of our study. A future approach to synchronization with moving visual stimuli under external vs. internal pacing might relate response taps to EEG indices of beat imagery (see Fujioka et al., 2015).

Concerning the effects of external vs. internal pacing on perception, we saw interactions with domain. However, since we did not see a powerful ball effect under external pacing (stimulus-guided), pacing effects in perception were less relevant to our goals than those found in synchronization.

Our study has a number of limitations, but it also affords new insights that motivate future research. One limitation relates to the equivalence between our synchronization and perception tasks. We cannot completely rule out that the dissociation between synchronization and perception was partly due to differences in stimulus structure and complexity in the two tasks, namely as far as the bouncing ball is concerned. Despite our efforts to shorten the trajectory when IOIs were shorter (300 ms), we may not have succeeded in eliminating the potential unnaturalness of fast-moving bouncing balls. It is also possible that the distance we used was too short given the ball’s size. Although we advocate that future research should work on novel solutions for this task-equivalence problem, other results of our study strengthen the possibility of dissociation between perception and synchronization. First, participants showed above-chance discrimination of stimulus-guided balls, suggesting that perceptual performance for balls was not totally impaired. Second, performance in synchronization did not correlate with performance in the perceptual task for any stimulus type. So, if there were detrimental effects of stimulus structure and complexity, these were not limited to balls.

The powerful ball effect we saw in stimulus-guided synchronization is noteworthy. Our effect was a strong one, and it met our highest expectations. The fact that our linear-trajectory balls were able to match beeps in synchronization raises novel hypotheses on the properties that are critical for enhancing visual stimuli in synchronization. Although previous research suggested that rectified trajectories, gravity and smoothness features could determine whether or not balls would match beeps (Hove et al., 2013b; Gan et al., 2015; Iversen et al., 2015), our results indicate that may not be entirely accurate. It seems possible that balls achieve maximum efficiency (“power”) with a linear trajectory, provided that fully continuous movement occurs and the impression of collision is formed. Previous studies have simulated collision points by representing the ground with a horizontal line (Hove et al., 2013b; Gan et al., 2015; Iversen et al., 2015). We used the alternative approach of squashing the ball as it hit the ground. Since we did not compare the two approaches (squashing vs. line), we do not know which is more efficient. Future research may address this topic.

The effects of internal pacing (imagery) on synchronization are potentially relevant to the field of neurological rehabilitation. Synchronization with a beat has been increasingly used in the rehabilitation of Parkinson’s disease (Nombela et al., 2013; Repp and Su, 2013; Schaefer, 2014), and the potential of imagery-guided synchronization is gaining increased attention (Satoh and Kuzuhara, 2008; Schaefer et al., 2014) adding to ongoing questions on modality (Hove and Keller, 2015). Concerning the possibility of using imagery, our findings afford different predictions depending on whether we focus on phase-matching (variability in asynchronies) or period-matching (IRI variability). The results for asynchrony suggest that beeps may be more effective than sirens in conditions of internal pacing. Also according to our results, the efficiency of all types of external pacers should decrease when they are used as internal pacers, and beeps should be particularly sensitive to the deleterious effects of imagery (stimulus removal). The results for period-matching suggest a different picture, according to which the efficacy of all stimuli may remain unchanged after stimulus removal. According to Thaut, who first proposed synchronization-based therapy for Parkinson’s disease, period-matching is the relevant goal to achieve (Thaut et al., 2015), so maybe there are reasons to be optimistic about rehabilitation programs using imagery-based synchronization. In any case, one should be extremely cautious about these predictions because we measured finger tapping instead of gait, and it is known that these movements involve different requirements (Repp and Su, 2013). Mostly, we should be cautious because we analyzed the responses of healthy individuals, and it is also known that Parkinson’s disease patients are vulnerable to certain task demands (Hove et al., 2012).

In the perceptual task, we saw a detrimental effect of imagery (internal pacing) on the bouncing ball, in that participants discriminated between correct and incorrect ball rhythms in the stimulus-guided domain, but responded by chance when imagery was introduced. Our results have pointed to the particular vulnerability of balls in driving internal pacing in the context of a perceptual task, and it may be interesting to find out which of the non-shared processes between perception and imagery play a role in this.

Also in the perceptual task, the response to different deviation types mainly reflected modality effects. We approached this as evidence that perception does not show the powerful ball effect (modality × continuity interaction), but the specific pattern of findings raises new interesting research questions too. In the stimulus-guided domain, we found that the visual modality benefits from violations in the length of two successive intervals compared to a single one (preference for type 2 compared to type 1 deviations), while the auditory modality shows no preference. One reason for this may be that visual perception engages narrower time windows, and the contrast between two successive deviated intervals with opposite tendencies (enlarged-diminished or vice-versa) as it happens in type 2 deviations may be easier to detect. Differently, audition may focus on broader time windows, relying more on beat representations that have been acquired earlier and thus less dependent on the extreme contrast between successive intervals as it happens in type 2 deviations. In the imagery-guided domain, auditory continuous stimuli (sirens) benefitted from incorrect intervals with on-time onsets (type 1) compared to intervals with off-time onsets. So, when dealing with sirens, participants did not favor onset-cues (absolute expectations, violated by type 2-deviations) after imagery. One reason for this may be the lack of salience of the beat onset in sirens (continuous stimuli). It could be argued that balls are also continuous, and thus balls should also discard onset-cues. However, balls present an anchor point when hitting the ground and squashing, which is likely a better onset-cue than the pitch maximum in siren pitch-curves.

Our findings contributed to better understand modality effects on beat-based temporal perception. Another interesting topic for future research may be how those modality effects operate in duration-based timing (Teki et al., 2011).

## Conclusion

Our study was novel in testing the powerful ball effect in a purely perceptual task, as well as under internal pacing conditions. Our findings suggest that the advantages of moving visual stimuli over static ones relate to action rather than perception, and they support the hypothesis that the sensorimotor coupling mechanisms for auditory (beeps) and moving visual stimuli (bouncing balls) overlap. Our findings contribute to expand the emergent evidence for dissociations between rhythmic skills (Tierney and Kraus, 2015) and between perception and action in temporal processing (Fujii and Schlaug, 2013).

## Author Contributions

SS conceived the experiment, collected, analyzed and interpreted data, wrote a draft, approved the final version of the draft and is accountable for all aspects of the work. SLC conceived the experiment, revised the draft for important intellectual content, approved the final version of the draft and is accountable for all aspects of the work.

## Conflict of Interest Statement

The authors declare that the research was conducted in the absence of any commercial or financial relationships that could be construed as a potential conflict of interest.

## Footnotes

1 https://www.pstnet.com/eprime.cfm

2 http://www.fon.hum.uva.nl/praat/

3 www.mathworks.com
