# Supplementary material for: Moving Stimuli Facilitate Synchronization But Not Temporal Perception
Source: Front Psychol. 2016 Nov 17;7:1798. doi: 10.3389/fpsyg.2016.01798 (PMC5112270; doi:10.3389/fpsyg.2016.01798)
Supplement: Supplementary file 1 [file Table_1.docx]

Supplementary Material

**How powerful is the ball? Moving stimuli require action but not sensory processes to facilitate temporal performance in the visual domain**

Susana Silva*, São Luís Castro*

*** Correspondence:** Susana Silva, Neurocognition and Language Research Group, Center for Psychology at University of Porto, Faculty of Psychology and Educational Sciences, University of Porto, Rua Alfredo Allen, 4200-135, Porto, Portugal, [susanamsilva@fpce.up.pt](mailto:susanamsilva@fpce.up.pt) and São Luís Castro, Neurocognition and Language Research Group, Center for Psychology at University of Porto, Faculty of Psychology and Educational Sciences, University of Porto, Rua Alfredo Allen, 4200-135, Porto, Portugal, [slcastro@fpce.up.pt](mailto:slcastro@fpce.up.pt)

# Supplementary Data

APPENDIX - Stimulus sequences (IOIs) for error detection task

|  | **Error type** | **Intervals correct version (ms)** | **Intervals incorrect version (ms)** |
| --- | --- | --- | --- |
| 1 | Type 1 | **600-600-600**-600-  600-600-600-600-  300-**300-(600)** | **600-600-600**-600-  600-600-600-600-  300-***433*-(467)** |
| 2 | Type 1 | **600-600-600**-600-  600-600-300-**300-(600)** | **600-600-600**-600-  600-600-300-***167*-(733)** |
| 3 | Type 1 | **600-600-600**-600-  600-600-600-300-**300-(600)** | **600-600-600**-600-  600-600-600-300*-****433*-(467)** |
| 4 | Type 1 | **600-600-600**-600-  600-300-**300-(600)** | **600-600-600**-600-  600-300-***167*-(733)** |
| 5 | Type 2 | **600-600-600**-600-  600-600-600-600-  600-**300-(300)** | **600-600-600**-600-  600-600-600-600-  *467*-***433*-(300)** |
| 6 | Type 2 | **600-600-600**-600-  600-600-600-600-  **300-(300)** | **600-600-600**-600-  600-600-600-*733*-  ***167*-(300)** |
| 7 | Type 2 | **600-600-600**-600-  600-600-600-**300-(300)** | **600-600-600**-600-  600-600-*467-****433*-(300)** |
| 8 | Type 2 | **600-600-600**-600-  600-600-**300-(300)** | **600-600-600**-600-  600-*733-****167*-(300)** |

Numbers in bold indicate the intervals that are preserved in imagery-based versions, and the ones in italics are those that were changed in incorrect versions. The final interval is indicated in parenthesis, since it has an undefined end point (the end of the stimulus).

**
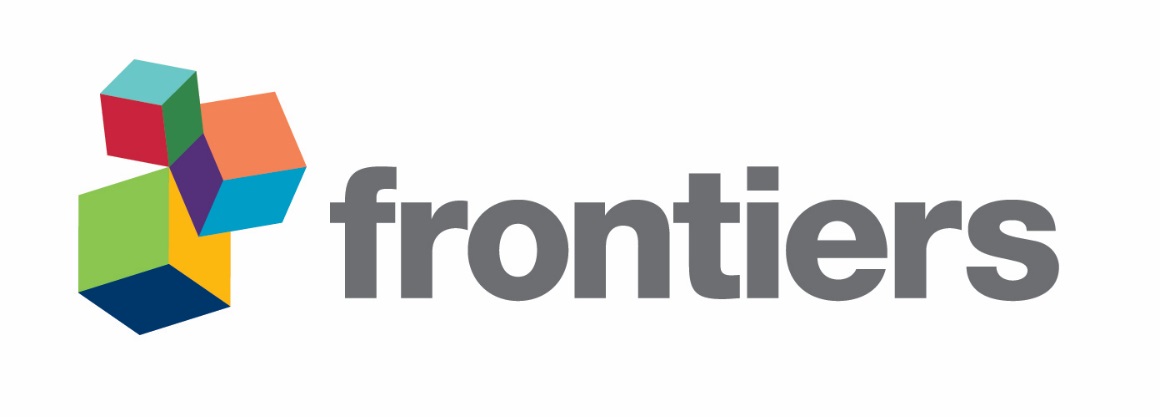
**
